# Restricted economic activity due to health conditions and risk of depression: results from the Korean Longitudinal Study of Aging

**DOI:** 10.3389/fpubh.2025.1442925

**Published:** 2025-02-18

**Authors:** Il Yun, Jae-Hyun Kim, Jong Youn Moon

**Affiliations:** ^1^Department of Preventive Medicine, Gachon University College of Medicine, Incheon, Republic of Korea; ^2^Department of Health Administration, College of Health Science, Dankook University, Cheonan, Republic of Korea; ^3^Artificial Intelligence and Big-Data Convergence Center, Gil Medical Center, Gachon University of Medicine, Incheon, Republic of Korea

**Keywords:** restricted economic activity, depression, health conditions, longitudinal study, CESD-10

## Abstract

**Purpose:**

This study aimed to investigate the association between the restricted economic activity due to health conditions and risk of depression, and further evaluate the differences in this association according to gender and across the older people.

**Methods:**

Data from the KLoSA from 2006 to 2016 was used and 10,144 research samples were included at baseline at 2006. Generalized estimating equation (GEE) model was applied in this longitudinal analysis.

**Results:**

Of the 10,144 individuals at baseline 2006, the odds ratio (OR) of depressive symptoms in those responding “very probable” on restricted economic activity was 2.88 times higher (*p*-value: <0.0001) compared with “not at all” respondents of restricted economic activity. In 64 years or less and 65 years or more, OR of depressive symptoms in “very probable” respondents of restricted economic activity was 3.03 times higher (*p*-value: <0.0001) and 2.85 times higher (*p*-value: <0.0001) compared with “not at all” respondents of restricted economic activity, respectively. In male and female, OR of depressive symptoms in “very probable” respondents of restricted economic activity was 1.76 times higher (*p*-value: <0.0001) and 1.56 times higher (*p*-value: <0.0001) compared with “not at all” respondents of restricted economic activity, respectively.

**Conclusion:**

This study demonstrated that restricted economic activity due to adverse health conditions was associated with risk of depression, especially among men between the ages of 45 ~ 64. These results suggest that in order to improve mental health in the middle-aged people, public health interventions are needed to sustain economic activity.

## Introduction

Clinical depression presents a significant challenge for adults because it is associated with lower quality of life, poor work performance, stress in social relationships (1) and a higher risk of developing psychiatric disorders (1). Although, the primary factors identified as the cause of these depressive symptoms may be genetic (2) and neurobiological (3), socioeconomic status such as education, (4) poverty (5) and employment status (6) and health behaviors (5) such as physical activity, smoking status and chronic disease is associated with higher risk of depression.

In general, working enables an individual to experience improved self-esteem and self-identity, in addition to being satisfied for being able to provide for the household. In addition, it seems to be potentially protective against chronic courses of depression over the lifetime (7), mainly by promoting independence and material well-being, through work salary, as well as the social benefits of paid work (6). Also, additional factors such as “feeling useful,” “empowered” or “having good social networks” have recently been identified (7). Furthermore, in a previous study, social participation and economic activity were associated with improvements in private life and health of the older people (8). Notably, the association between social network and health has shown to be bidirectional, in which poor health could restrict social activity (9), or the social capital of older adults could affect their health, either directly or indirectly (10). Also, the index of older people’s economic activity is closely associated with their health status. For example, among retired older people, work limitation was related to poor health status (11).

Currently, South Korea is facing new challenges in the labor market, such as a rise in labor inequalities and informal employment, weak recovery of salaries, and persistent need for greater participation of the older adults’ labor force. The older adults’ labor dynamics also require the integration of older adults’ perspective into the study of mental health and employment. Various studies have been conducted on the effects of unemployment on depression (12–14). However, on the contrary, there are studies showing that unemployment occurs in people with depressive symptoms as they become older adults (15, 16). Using longitudinal data, we attempted to find out the temporal relationship of restriction on economic activity due to health condition leads to increases depressive symptoms in older people.

Therefore, the objectives of this analysis are: (1) to estimate the association between the restricted economic activity due to health condition and depressive symptoms in middle aged and older people; (2) to evaluate the differences in this association between men and women, and across the older people.

## Methods

### Data source

The data used for the following analyses were derived from the Korean Longitudinal Study of Aging (KLoSA) from 2006 to 2016. As a type of study that possesses both the strengths of cross-sectional data and time series data, the KLoSA was constructed by repeatedly surveying the identical content for the same respondents every year. Thus, all variables surveyed by the KLoSA were repeatedly measured from the 1st wave to the 4th wave to collect observation cases at multiple points in time. This biennial survey involves multistage stratified sampling based on geographical areas and housing types across Korea. Participants were selected randomly using a multistage, stratified probability sampling design to create a nationally representative sample of community-dwelling Koreans 45 years of age and older. Participant selection was performed by the Korea Labor Institute, including individuals from both urban and rural areas. In case of refusal to participate, another participant was selected from an additional, similar sample from the same district. We accessed the KLoSA dataset in March 21, 2022 for research purposes.

In the first baseline survey in 2006, 10,254 individuals in 6,171 households (1.7 per household) were interviewed. There were 292 individuals with cancer. The second survey, in 2008, followed up with 8,675 participants, who represented 86.6% of the original panel. The third survey, in 2010, followed up with 8,229 participants, who represented 81.7% of the original panel, the fourth survey, in 2012, followed up with 7,813 participants, who represented 80.1% of the original panel and the fifth survey, in 2014, followed up with 8,387 participants (including 920 new participated sample), who represented 80.4% of the original panel. The sixth survey, in 2016, followed up with 9,913 (including 878 new participated sample), who represented 79.6% of the original panel.

**Figure 1:**
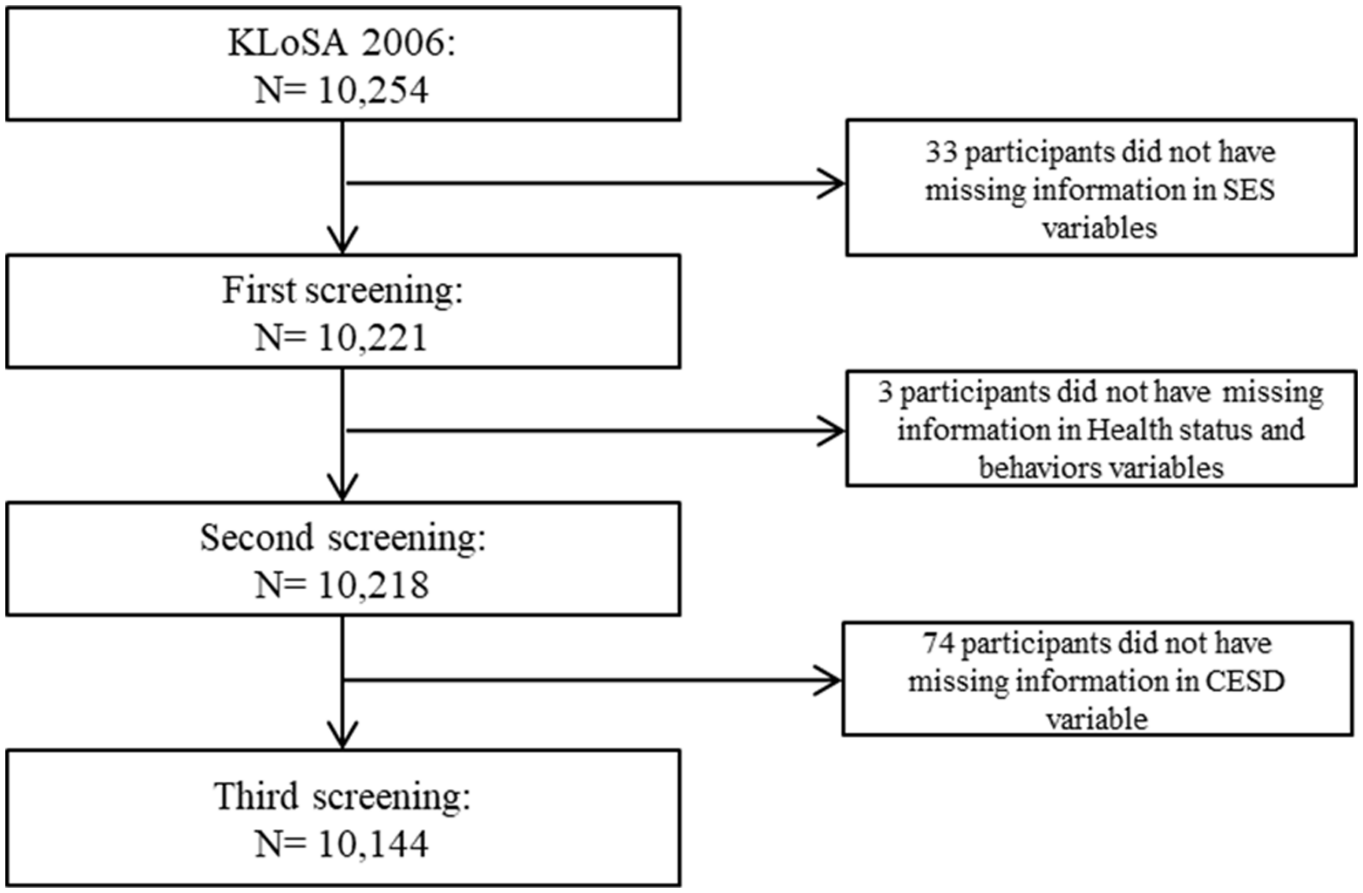
Flow chart for sample selection.

Incomplete data was excluded: 33 individuals who lacked information on socioeconomic factors and 23 individuals who lacked information on health status and risk factors in 2006. Finally, 10,144 research samples were included (Figure 1). Because this study used KLoSA which is a public open database, anonymity is guaranteed and it is not a human-derived study and trained investigators was given informed consent from individuals who wished to voluntarily participate in the study, institutional review board (IRB) is not necessary.

### Independent variables

Restriction on economic activity due to health condition was assessed by self-reported response to the question: “Do you have a problem with your work because of your health condition?” the responses were assigned to 1 of 4 subcategories: “very probable,” “probable,” “probably not” and “not at all.”

### Dependent variables

#### Depressive symptoms

Self-reported data regarding depressive symptoms were extracted from responses to the question: “Do you think you were depressed for a week?” the response “less than a day” indicated “No” and “a day or two,” “three or four day” and “five days or more” indicated “Yes” thus dichotomizing the response for multiple logistic analysis.

#### Depression – CESD 10

The 10-item version of the Center for Epidemiologic Studies Depression (CES-D) scale was used. This instrument has proven to be a useful indicator of depression among older adults (17, 18, 19). The CESD-10 scale has shown good predictive accuracy when compared with its full-length 20-item version. The brief CES-D scale consists of 10 items assessing three factors; depressed affect (feeling blue, depressed, fear, loneliness), psychomotor retardation (irritability, sleep difficulties, decreased energy, and problems with attention), and positive affect (happy, hopeful). The time frame for assessing depressive disorder was 7 days prior to the interview. CESD-10 scale was used as a continuous measure.

### Control variables

#### Socioeconomic and demographic factors

Age groups were divided into three categories: 45–54, 55–64 and ≥ 65 years of age. Education level was categorized into four groups: elementary school or lower, middle school, high school, and college or higher. Gender was categorized as male and female. Residential regions were categorized as Metropolitan (Seoul), urban (Daejeon, Daegu, Busan, Incheon, Kwangju, or Ulsan) or rural (not classified as a city). Marital status was divided into three groups: married, separated or divorced, and single. Health insurance was categorized into national health insurance and medical aid.

#### Health status and behavioral factors

Smoking status was categorized into three groups: current smoker, former smoker, and never smoker. Alcohol use was also divided into three groups: current drinker, former drinker, and never drinker. Self-rated health was categorized into five groups: very good, good, normal, bad, and worst. Finally, the number of chronic diseases (consisting of hypertension, diabetes, osteoarthritis, rheumatoid arthritis, cancer, chronic pulmonary disease, liver disease, cardiovascular disease, and cerebrovascular disease) and year dummies were included as covariates in our analyses.

#### Analytical approach and statistics

Chi-square test and a generalized estimating equation (GEE) model were used. The use of a GEE model was required in order to handle the unbalanced data with correlated outcomes over time. To determine whether the probability of depression changed over time, time (year) was included in the model as a categorical covariate; the regression coefficient was used to estimate both the change in probability of depression and independent variables, annually (20). For all analyses, statistical significance was set to *p* ≤ 0.05, two-tailed. All analyses were conducted using the SAS statistical software package, version 9.4 (SAS Institute Inc., Cary, NC, USA).

## Results

### Prevalence of depression

Table 1 displays the descriptive statistics of all variables at baseline (2006). Of the 10,144 research subjects included in our study, the prevalence of depressive symptoms was 26.2% (26.2 participants) (Table 1) and mean of CESD was 3.01 points (SD: 2.69). Of the total sample population, 62.6% (*n* = 704 participants) of the participants with depressive symptoms responded “very probable” on restriction on economic activity due to health condition, and 37.2% (*n* = 864 participants) of the participants with depressive symptoms responded “probable” on restriction on economic activity due to health condition. In terms of CESD, CESD of those responding “very probable” on restriction on economic activity due to health condition was 5.72 points (SD: 2.92) and CESD of those responding “probable” on restriction on economic activity due to health condition was 3.78 points (SD: 2.84).

**Table 1:** General characteristics of subjects included for analysis.

	Total		Depressive symptom		*p*-value	CESD		*p*-value
	*N*	%	Yes	%		Mean	SD	
Restriction on economic activity due to health condition					0.0001			<0.0001
Very probable	1,124	11.1	704	62.6		5.72	2.92	
Probable	2,326	22.9	864	37.2		3.78	2.84	
Probably not	4,183	41.2	753	18		2.47	2.25	
Not at all	2,511	24.8	336	13.4		1.99	2.05	
Education level					<0.0001			<0.0001
≤ Elementary school	4,751	46.8	1,748	36.8		3.79	2.91	
Middle school	1,644	16.2	385	23.4		2.73	2.49	
High school	2,695	26.6	397	14.7		2.23	2.2	
≥ College	1,054	10.4	127	12.1		1.96	2.02	
Gender					<0.0001			<0.0001
Male	4,426	43.6	902	20.4		2.6	2.48	
Female	5,718	56.4	1,755	30.7		3.33	2.81	
Age					<0.0001			<0.0001
≤54	3,268	32.2	541	16.6		2.27	2.26	
55–64	2,769	27.3	640	23.1		2.71	2.55	
≥65	4,107	40.5	1,476	35.9		3.8	2.89	
Marital status					<0.0001			<0.0001
Married	7,912	78	1,682	21.3		2.65	2.49	
Separated, divorced	2,146	21.2	945	44		4.31	2.99	
Single	86	0.9	30	34.9		3.84	2.9	
Residential region					0			0.003
Metropolitan	1,737	17.1	416	24		3.06	2.71	
Urban	2,946	29	717	24.3		2.85	2.63	
Rural	5,461	53.8	1,524	27.9		3.08	2.72	
Health insurance					<0.0001			<0.0001
National health insurance	9,522	93.9	2,323	24.4		2.87	2.6	
Medical aid	622	6.1	334	53.7		5.22	3.07	
Self-rated Health					<0.0001			<0.0001
Very Good	355	3.5	28	7.9		1.73	1.63	
Good	3,487	34.4	340	9.8		1.82	1.81	
Normal	3,180	31.4	687	21.6		2.7	2.34	
Bad	2,444	24.1	1,121	45.9		4.37	2.91	
Worst	678	6.7	481	70.9		6.37	2.7	
Number of chronic disease*					<0.0001			0.963
0	5,398	53.2	1,002	18.6		2.43	2.34	
1	2,936	28.9	874	29.8		3.3	2.8	
≥2	1,810	17.8	781	43.2		4.26	2.98	
Smoking status					0.003			0.021
Never	7,217	71.2	1,957	27.1		3.08	2.71	
Former smoker	973	9.6	224	23		2.92	2.57	
Smoker	1,954	19.3	476	24.4		2.78	2.67	
Alcohol use					<0.0001			0.4
Drinker	9,464	93.3	2,417	25.5		2.95	2.66	
Former Drinker	680	6.7	240	35.3		3.81	2.97	
Never								
Total	10,144	100	2,657	26.2		3.01	2.69	

^*^Hypertension, diabetes, cancer, chronic obstructive pulmonary disease, liver disease, cardiovascular disease, cerebrovascular disease, arthritis.

### Association between restriction on economic activity and depression

Table 2 shows the relationship between the restriction on economic activities due to health conditions and risk of depression adjusted for socioeconomic status and health risk status and behavior factors. After adjusting for all of these confounders, the odds ratio (OR) of depressive symptoms in those responding “very probable” on restriction on economic activity due to health condition was 2.88 times higher (95% Confidence Interval [CI]: 2.58–3.22 *p*-value: <0.0001) compared with those responding “not at all” on restriction on economic activity due to health condition. In terms of CESD, after adjusting for all confounders, the estimates for CESD was 0.50 higher (95% CI: 0.46–0.54 *p*-value: <0.0001) in “very probable” and 0.39 higher (95% CI: 0.36–0.43 *p*-value: <0.0001) in “probable” respondents, compared to “not at all” respondents of restriction on economic activity due to health condition.

**Table 2:** Adjusted effect between economic activity and depression.

	Depressive symptom				CESD			
	OR	95% CI		*p*-value	*B*	95% CI		*p*-value
Restriction on economic activity due to health condition								
Very probable	2.88	2.58	3.22	<0.0001	0.50	0.46	0.54	<0.0001
Probable	2.03	1.88	2.20	<0.0001	0.39	0.36	0.43	<0.0001
Probably not	1.51	1.41	1.63	<0.0001	0.26	0.22	0.29	<0.0001
Not at all	1.00				ref			
Education level								
≤ Elementary school	1.35	1.24	1.48	<0.0001	0.13	0.10	0.16	<0.0001
Middle school	1.27	1.16	1.39	<0.0001	0.10	0.07	0.13	<0.0001
High school	1.17	1.07	1.27	0.00	0.07	0.03	0.10	<0.0001
≥ College	1.00				ref			
Gender								
Male	0.89	0.84	0.95	0.00	−0.04	−0.06	−0.02	<0.0001
Female	1.00				ref			
Age								
45–54	1.00				1.00			
55–64	0.99	0.92	1.06	0.80	0.00	−0.03	0.02	0.94
≥65	1.07	0.99	1.15	0.08	0.06	0.03	0.08	<0.0001
Marital status								
Married	0.60	0.47	0.76	<0.0001	−0.14	−0.21	−0.08	<0.0001
Separated, divorced	0.93	0.73	1.18	0.54	−0.01	−0.08	0.05	0.67
Single	1.00				ref			
Residential region								
Metropolitan	1.00				ref			
Urban	0.99	0.92	1.05	0.69	−0.07	−0.09	−0.05	<0.0001
Rural	1.13	1.06	1.20	<0.0001	0.00	−0.02	0.02	0.91
Health insurance								
National health insurance	1.00				ref			
Medical aid	1.46	1.33	1.59	<0.0001	0.11	0.09	0.13	<0.0001
Self-rated Health								
Very Good	1.00				ref			
Good	1.51	1.24	1.85	<0.0001	0.06	−0.02	0.14	0.12
Normal	2.30	1.88	2.81	<0.0001	0.23	0.16	0.31	<0.0001
Bad	4.52	3.69	5.54	<0.0001	0.48	0.41	0.56	<0.0001
Worst	10.42	8.30	13.08	<0.0001	0.69	0.61	0.77	<0.0001
Number of chronic disease*								
0	1.00				ref			
1	0.92	0.87	0.98	0.01	−0.03	−0.05	−0.01	0.00
≥2	0.97	0.92	1.02	0.22	−0.02	−0.03	0.00	0.01
Smoking status								
Never	0.89	0.83	0.95	0.00	−0.04	−0.06	−0.02	0.00
Former smoker	0.86	0.80	0.93	0.00	−0.03	−0.06	−0.01	0.01
Smoker	1.00				ref			
Alcohol use								
Drinker	0.94	0.84	1.05	0.26	−0.02	−0.05	0.02	0.32
Former drinker	0.95	0.89	1.02	0.13	−0.03	−0.05	−0.01	0.00
Never	1.00				ref			
Year								
2006	0.58	0.54	0.63	<0.0001	0.11	0.09	0.14	<0.0001
2008	1.20	1.11	1.29	<0.0001	0.31	0.28	0.33	<0.0001
2010	1.10	1.02	1.19	0.01	0.28	0.25	0.30	<0.0001
2012	0.99	0.92	1.07	0.80	0.24	0.21	0.26	<0.0001
2014	1.06	0.96	1.17	0.25	0.13	0.10	0.16	<0.0001
2016	1.00				ref			

### Association between restriction on economic activity and depression by age and gender

Table 3 shows subgroup analysis according to age (64 years or less and 65 years or more) and gender (male and female). In 64 years or less, OR of depressive symptoms in those responding “very probable” on restriction on economic activity due to health condition was 3.03 times higher (95% CI: 2.49–3.69 *p*-value: <0.0001) and in 65 years or more, OR of depressive symptoms in those responding “very probable” on restriction on economic activity due to health condition was 2.85 times higher (95% CI: 2.44–3.34 *p*-value: <0.0001) compared with those responding “not at all” on restriction on economic activity due to health condition. In the male group, OR of depressive symptoms in those responding “very probable” on restriction on economic activity due to health condition was 1.76 times higher (95% CI: 1.67–1.87 *p*-value: <0.0001), and in the female group, OR of depressive symptoms in those responding “very probable” on restriction on economic activity due to health condition was 1.56 times higher (95% CI: 1.49–1.63 *p*-value: <0.0001) compared with those responding “not at all” on restriction on economic activity due to health condition. In terms of CESD of 64 years or less, the estimates for CESD was 0.56 higher (95% CI: 0.51–0.62 *p*-value: <0.0001) and in 65 years or more, the estimates for CESD was 0.44 higher (95% CI: 0.38–0.49 *p*-value: <0.0001) in “very probable” respondents of restriction on economic activity due to health condition, compared to “not at all” respondents of restriction on economic activity due to health condition. In the male group, the estimates for CESD was 0.57 higher (95% CI: 0.51–0.62 *p*-value: <0.0001) and in the female group, the estimates for CESD was 0.44 higher (95% CI: 0.40–0.49 *p*-value: <0.0001) in “very probable” respondents of restriction on economic activity due to health condition, compared to “not at all” respondents of restriction on economic activity due to health condition.

**Table 3:** Adjusted effect between economic activity and depression by gender and age.

	Depressive symptom				CESD			
	OR	95% CI		*p*-value	*B*	95% CI		*p*-value
Restriction on economic activity due to health condition	≤64							
Very probable	3.03	2.49	3.69	<0.0001	0.56	0.51	0.62	<0.0001
Probable	2.19	1.96	2.45	<0.0001	0.46	0.42	0.5	<0.0001
Probably not	1.43	1.31	1.57	<0.0001	0.25	0.21	0.29	<0.0001
Not at all	1.00				ref			
Restriction on economic activity due to health condition	≥65							
Very probable	2.85	2.44	3.34	<0.0001	0.44	0.38	0.49	<0.0001
Probable	1.99	1.75	2.26	<0.0001	0.33	0.27	0.38	<0.0001
Probably not	1.59	1.41	1.8	<0.0001	0.22	0.17	0.27	<0.0001
Not at all	1.00				ref			
Restriction on economic activity due to health condition	Male							
Very probable	1.76	1.67	1.87	<0.0001	0.57	0.51	0.62	<0.0001
Probable	1.58	1.5	1.66	<0.0001	0.46	0.41	0.51	<0.0001
Probably not	1.34	1.28	1.41	<0.0001	0.29	0.25	0.34	<0.0001
Not at all	1.00				ref			
Restriction on economic activity due to health condition	Female							
Very probable	1.56	1.49	1.63	<0.0001	0.44	0.4	0.49	<0.0001
Probable	1.41	1.35	1.47	<0.0001	0.34	0.3	0.38	<0.0001
Probably not	1.24	1.19	1.29	<0.0001	0.21	0.17	0.25	<0.0001
Not at all	1.00				ref			

## Discussion

In this population-based study of 10,144 middle aged and older adults at baseline, our primary purpose was to investigate whether the restriction on economic activity due to health condition was responsible for the depressive symptom and CESD after adjusting for covariates, including socioeconomic status and health risk and behavior factors, using nationally representative database in South Korea. In the present study, our results presented an association between the restriction on economic activity due to health and depression, especially for those aged 64 years or less.

The economic activity was influenced by health status, which was better for those who were employed and had a large income (21). It is plausible to consider that people with good socioeconomic status have positive expectations regarding their health (22), consequently contributing to their subjective health. According to Dweyer DS et al. (23), the functional limitation level could be used as a sufficient objective proxy of the underlying health status in economic activities, and self-reported work limitations could perform similarly as a subjective measure. Previous studies showed associations between health, mediated by economic activities, and life satisfaction (24), the individual’s social network (25), as well as social participation (26). In another study, an association between the health condition and indices of economic activity (e.g., income and property ownership) was found (27). In a study by Dwyer DS et al., they disaggregated reported health conditions and analyzed their effects on retirement. Also, where some chronic conditions (e.g., functional limitations and circulatory disorders) accelerated retirement, conditions such as nervous disorders and injuries did not.

Social geriatricians often use the activity theory to explain the association between activities and mental health. According to the activity theory, interpersonal activities are beneficial for psychological well-being, which is mainly due to the functional support provided by social integration, and gives meaning to later life (28). Additionally, Herzog and House (29) reported that by participating in various activities (e.g., paid work, self-care, volunteer work and informal assistance), older adults can contribute socially. Furthermore, in a study conducted by Schwingel, (13), being able to stay economically active after retirement for people aged 55 years and older was beneficial in terms of their mental health.

The results of our study reported that the association between economic activities and depression varied according to age and gender. Although the prevalence of depression in women was higher than that of men, an association between restricted economic activity and the depression was more pronounced in 64 years old or younger male adults, which is similar with results from a previous study (30). It is possible that the gender differences in this association between economic activities and depression may be the result of the Confucian culture of Korea. In Korea, the burden of social production tends to rest more on men than for women. In addition, Kim et al. (31) indicated that women’s subjective health was more influenced by demographic status than economic activities, because the results of this study presented higher subjective health status in females compared to their counterparts.

The purpose of this study was to investigate differences in the prevalence of depression by restricted economic activity due to health condition, taking into account the general characteristics that can affect depression. This finding seems to be similar to previously reported research showing differences in the associations between economic activity and mental health. These studies show that economic activity is more closely related to mental health in men than in women.

Economic activities of the middle aged and older adults are an important factor to satisfy their various desires, including alleviating poverty, improving quality of life, and fostering activities of daily living and health. Therefore, in South Korea which is facing new challenges in the labor market such as rise in labor inequalities and informal employment, the economic activity associated with mental health is an important social issue for increasingly aging workers.

Scientific evidence about these issues will help governments develop public health, economic, and social policies to urge those with restricted economic activity to improve their health condition. Thus, they should create more opportunities for women in the labor market and encourage men to share the domestic work. In addition, in order to prevent the increase in social burden caused by depression due to unemployment, specific interventions such as encouraging labor activities that can be done even by those in poor health will be necessary.

There are several limitations to this study that should be taken into consideration. First, data was gathered from self-reports of socioeconomic factors as well as health status and risk factors. Self-reporting data may be an imperfect indicator of actual behavior. Second, information regarding health status and risk behavior factors was not sufficient. Furthermore, there might have been unobserved confounders. For example, social support or caregiving may have an important impact on the mental health of people whose economic acuity is limited due to poor health conditions, but there was no variable measuring these in the data used. The lack of such information might have resulted in an underestimation of our results in the present study. Nevertheless, despite the underestimation, we found a significant association between restricted economic activity and depression. Future studies should include all potential confounders to enable more precise association estimates.

Despite these limitations, this study has various strengths, particularly with its use of a population-based representative sample and the 10-year follow-up database. We also prospectively analyzed a large number of individuals from longitudinal data of a well-defined and comprehensively studied sample of middle aged and older adults to examine the association between restricted economic activity and depression. Therefore, with the rapidly aging population in Korea, restricted economic activity may be a reasonably good predictor of depression.

## Conclusion

In conclusion, restrictions on economic activities due to adverse health conditions are associated with depression, especially among community-dwelling men between the ages of 45 and 64 years. These results suggest that in order to improve mental health, public health interventions are needed to ensure that they continue to engage in economic activity.

## Data Availability

Publicly available datasets were analyzed in this study. This data can be found here: the dataset analyzed in the present study is publicly accessible. Available online: https://survey.keis.or.kr/klosa/klosa01.jsp.
